# Assessing the feasibility of introducing health insurance in Afghanistan: a qualitative stakeholder analysis

**DOI:** 10.1186/s12913-017-2081-y

**Published:** 2017-02-22

**Authors:** Wu Zeng, Christine Kim, Lauren Archer, Omarzaman Sayedi, Mohammad Yousuf Jabarkhil, Kathleen Sears

**Affiliations:** 1 Schneider Institutes for Health Policy, Heller School, Brandeis University, MS 035, Waltham, MA 02454-9110 USA; 2 Gillings School of Global Public Health, University of North Carolina at Chapel Hill, Chapel Hill, NC USA; 3 DKT International, Patna, Bihar India; 4 Palladium Group, Kabul, Afghanistan; 5 Palladium Group, Washington, DC USA

**Keywords:** Health insurance, Feasibility, Stakeholder analysis, Universal Health Coverage, Afghanistan

## Abstract

**Background:**

In the last decade, the health status of Afghans has improved drastically. However, the health financing system in Afghanistan remains fragile due to high out-of-pocket spending and reliance on donor funding. To address the country’s health financing challenges, the Ministry of Public Health investigated health insurance as a mechanism to mobilize resources for health. This paper presents stakeholders’ opinions on seven preconditions of implementing this approach, as their understanding and buy-in to such an approach will determine its success.

**Methods:**

Key informant interviews and focus group discussions were conducted with stakeholders. The interviews focused on perceptions of the seven preconditions of introducing health insurance, and adapting a framework developed by the International Labor Organization. Content analysis was conducted after interviews and discussions were transcribed and coded.

**Results:**

Almost all of the stakeholders from government agencies, the private sector, and development partners are interested in introducing health insurance in Afghanistan, and they were aware of the challenges of the country’s health financing system. Stakeholders acknowledged that health insurance could be an instrument to address these challenges. However, stakeholders differed in their beliefs about how and when to initiate a health insurance scheme. In addition to increasing insecurity in the country, they saw a lack of clear legal guidance, low quality of healthcare services, poor awareness among the population, limited technical capacity, and challenges to willingness to pay as the major barriers to establishing a successful nationwide health insurance scheme.

**Conclusions:**

The identified barriers prevent Afghanistan from establishing health insurance in the short term. Afghanistan must progressively address these major impediments in order to build a health insurance system.

**Electronic supplementary material:**

The online version of this article (doi:10.1186/s12913-017-2081-y) contains supplementary material, which is available to authorized users.

## Background

**Fig. 1:**
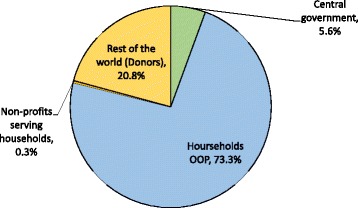
Financing sources of total health expenditure in Afghanistan, 2011–2012

Since 2001, significant improvements have been made in Afghanistan’s health sector. The Basic Package of Health Services (BPHS), which is free of charge and primarily supported by donors, has increased access to primary care services for the poor, especially for key maternal and child health services [1, 2]. Despite its achievements, service delivery challenges still exist, and the country continues to deal with a weak health financing system. The total health expenditure (THE) per capita increased from US$42 to US$56 between 2008–2009 and 2011–2012 [3]; compared to other countries in the global arena, however, this level of spending is low. Households’ out-of-pocket (OOP) expenditures or direct payments for health services are high, accounting for 73% of THE [3]. Moreover, Afghanistan is heavily donor dependent, with 75% of the total public health expenditure coming from external aid [3] (Fig. 1).

Similar to many post-conflict developing countries, Afghanistan has depended on external aid to reconstruct its health system over the past decade. It has a limited capacity for generating domestic revenues because the population is poor, the country has a largely informal economy, and collection of revenues for health through a broad tax base is weak [4, 5]. As external aid decreases and the country moves towards a model of more sustainable governance, alternative financing mechanisms will be crucial to the resilience of the existing health system and the health gains it has achieved.

Both the high OOP expenditure and high donor dependency in Afghanistan demand interventions to introduce financial risk protection mechanisms, such as health insurance, to move toward universal health coverage (UHC). Many developing countries in sub-Saharan Africa and Southeast Asia have launched ambitious national health insurance reforms to raise prepaid revenues for health, pool health risks, and purchase services more efficiently [6, 7].

Realizing challenges in many low- and mid-income countries to provide essential affordable quality care to populations, the World Health Organization (WHO) advocates the UHC agenda and further research on UHC [8–10]. One essence of UHC is to move away from direct payment to prepayment mechanisms as a means to improve access to care (both coverage of services and population) and to provide financial protection of the population seeking care. Given the high OOP expenditure and limited access to quality care, Afghanistan is in the very early stage of UHC, and needs policy changes and reforms to accelerate the pace towards UHC.

Experiences from other countries show that health financing reform and the introduction of health insurance are complex, long-term processes requiring strong technical and management capacity as well as political commitment from various stakeholders [11–13]. There is a growing recognition that health sector stakeholders have a strong influence on policy development and moving reforms forward in a country [14]. The inclusion of stakeholder perceptions, understanding, behaviors, interests, and intentions around health financing and health insurance will inform the feasibility of introducing health insurance schemes and the next steps to move forward. This is of particular importance in Afghanistan, where the health sector is shaped largely by donors as funders, nongovernmental organizations (NGOs) as providers, and the government as the steward, regulator and provider.

In an effort to address health financing challenges, the government of Afghanistan is interested in introducing national health insurance, which would primarily focus on secondary care, as a means of reducing the high financial burden on households and improving the country’s independence regarding the funding of healthcare. Meanwhile, health insurance is also expected to improve access to health care, particularly for vulnerable populations, and ultimately improve health status and equity of population health in Afghanistan. However, introducing health insurance is complex and requires a careful assessment of its feasibility. Many feasibility studies have only focused on financial viability of health insurance [15–17], and few take a holistic approach.

Guided by a feasibility assessment framework [18], this study aims to document stakeholders’ opinions on following seven preconditions for health insurance schemes in Afghanistan: (1) the need for health insurance, (2) political commitment, (3) legal and regulatory environment, (4) quality of care, (5) population awareness, (6) ability to pay, and (7) technical and operational capacity of health insurance. It should be noted that this study does not discuss the design of health insurance.

## Methods

### Study framework

To implement the feasibility assessment of introducing health insurance, we adapted the International Labor Office (ILO) framework developed for strategies and tools for the social exclusion and poverty global program (STEP) on microinsurance schemes [18] and are guided by the manual of planning social health insurance jointly developed by Asian Development Bank, ILO, WHO, and German Technical Cooperation. The feasibility of introducing health insurance was assessed by investigating the seven preconditions of establishing health insurance schemes, mentioned above.

### Study participants

We conducted a stakeholder assessment using both qualitative individual key informant interviews (KIIs) and focus group discussions (FGDs) to assess stakeholders’ interest in health insurance, and investigate preconditions of its introduction. In collaboration with the Health Economics and Financing Directorate (HEFD) at the Ministry of Public Health (MoPH), given HEFD’s extensive knowledge of the healthcare system, we identified 11 stakeholder groups for KIIs. KIIs were used to solicit information from selected individuals with high-level authority and/or special knowledge. KII stakeholders included high-level government officials from ministries and parliamentarians (i.e., the MoPH, Ministry of Finance [MoF], Ministry of Justice (MoJ), Ministry of Labor and Social Affairs [MoLSA], and Parliament); international donors and organizations (i.e., WHO, the European Commission [EC], USAID, and the World Bank); and private sector agencies (i.e., two private insurance companies).

We identified five stakeholder groups for participation in FGDs to document the perceptions, opinions, and attitudes toward the seven preconditions amongst those who perform similar functions in the healthcare system, such as those in the private sector and health service providers. These groups included (1) BPHS implementing managers; (2) public hospital directors; (3) private hospital directors; (4) organizations working on community savings projects; and (5) HEFD staff.

### Data collection

The KIIs and FGDs focused on different aspects of the health system as related to health insurance, depending on the participants’ organizational affiliations. For example, for the MoJ and Parliamentary survey participants—entities more familiar with regulations and the legal issues regarding health insurance—the interviews focused more on regulatory and legal constraints, and opportunities for implementing health insurance. For participants from private health insurance companies, the focus of the interviews was on understanding the insurance market, the operation of health insurance, and the capacity needed to implement or manage health insurance. For detailed interview guide, see Additional file 1.

Data were collected from July to August 2014, using semi-structured interview guides to frame questions and solicit stakeholders’ interests and opinions on the preconditions of health insurance. Interviewers explained the purpose of the study to all participants and obtained informed verbal consent at the beginning of each interview. They conducted all interviews in a quiet place.

The KIIs and FDGs lasted approximately 60 min and were conducted in the participants’ preferred language (English or Dari). Two experienced researchers conducted and facilitated a majority of the KIIs and FDGs, respectively. Both researchers are health financing experts with a strong understanding of the Afghan health system. They had translation assistance from national staff to conduct interviews in Dari, as needed. The translator had significant experience in working on health sector activities in Afghanistan and was trained by the researchers to ensure high-quality interviews and translations. In total, 22 KIIs and FGDs were conducted.

### Data analysis

All Interviews and discussions were digitally recorded and then transcribed for analysis in Atlas.ti qualitative analysis software. Three researchers analyzed the transcripts through direct content analysis, a qualitative data analysis method, using Atlas.ti software. Codes were developed primarily according to the seven themes (preconditions). Based on the initial review and the interview guide themes according to the ILO framework, we deductively organized a structure of initial code groups for analysis. Using this initial structure of codes, the researchers analyzed transcripts line by line and assigned new codes to additional emerging concepts. Atlas.ti was used to sort and organize the themes and to examine the patterns emerging from each of the themes. To ensure consistency and common understanding of code concepts, the analysts discussed the coding process and one senior member of the team checked the analysis results.

## Results

As mentioned in the Methods section, the stakeholder analysis identified seven key topics for assessment; below, we summarize and describe the interviews by topic.

### Perception of the need for health insurance

The majority of stakeholders were aware of the health financing situation in the country, which includes high OOP spending and reliance on donor funding, as well as the challenges of healthcare delivery. Stakeholders acknowledged that health financing reform (including the use of health insurance mechanisms) was needed to change the current situation and ensure a more sustainable health financing system for Afghanistan.

Although the government states that healthcare is a right for its citizens and reiterates its commitment to providing health services for the population, participants referred to the inability to provide financial protection as a key issue for the current system. People sometimes have to sell their assets or borrow from relatives and communities to seek healthcare in Afghanistan or other countries, such as India and Pakistan. Introducing health insurance could provide financial protection against catastrophic health expenditures, particularly for the poor.

“This [health insurance] should be our priority—to promote insurance services so that we could cover or bring more people under our insurance coverage.”

Participants also pointed out that health insurance also could help to improve the equity of healthcare in the country. The rising cost of health services due to new technology and changes in disease patterns has exacerbated inequality in the utilization of healthcare. Whereas the wealthy can obtain healthcare from private health facilities or go abroad, the poor have limited options. Health insurance could be an important tool for promoting greater equity of health service utilization.

### Leadership and political commitment

Healthcare reform and the successful implementation of health insurance schemes require strong political will and government stewardship, particularly in Afghanistan, where additional challenges exist due to insecurity, poverty, and numerous competing priorities.

“[Health insurance] will not be automatic because there are a lot of priorities. There is a need, I think, for some lobbying and advocacy. There should be some kind of agreement that a certain percentage [of government revenue] will be allocated for the social sector, especially for the health sector. Otherwise, the government always think[s] about infrastructure and security. Very often the social and health sector are left out.”

A majority of stakeholders acknowledged that strong leadership, multisectoral political commitment, and effective collaboration are essential to begin implementing health insurance in Afghanistan, but noted that the current political commitment should be strengthened, especially at the highest levels of government.

“… political will and political support are very important for establishing health insurance systems in every country. But besides this, for Afghanistan especially, I think it’s more important … because of the constitution, because of the law and the regulations we currently have, especially in terms of having all health services free. So we really need a very strong [political] will and support at different stages and in different entities of the government [to overcome the barriers].”

Stakeholders acknowledged that to be successful, advocacy efforts aimed at increasing support for and political commitment to health insurance must target leadership across different sectors and at different levels of government: “It requires a lot of lobbying with Parliament, the cabinet of Afghanistan, ministries, and the people.” Implementation of health reforms in Afghanistan also will require effective collaboration to design, implement, and manage a health insurance scheme.

### Legal and regulatory environment

Stakeholders identified the legal and regulatory conditions for health financing as major challenges that need to be addressed in the short term if a health insurance scheme is to be developed in the future. In particular, the ambiguity of Article 52, which addresses the state’s obligation to provide healthcare to its citizens, leaves the law open to misinterpretation. Article 52 states “The state is obliged to provide free means of preventive healthcare and medical treatment, and proper health facilities to all citizens of Afghanistan in accordance with the law” [19].

“According to the constitution, the health facilities are free for the people … it is clearly defined in … article 52 of [the] constitution: [paraphrase of constitution] ‘The government, in accordance to the law, provides preventive care, treatment, and health facilities free of charge to all the people’”.

Varying interpretations of the law and differing opinions exist—even among high-level government officials—about whether the law mandates that the government provide services for free to all Afghan citizens and what those services may include, or at what level. Whereas many understand Article 52 as a mandate for the provision of free services, some interpret it as only requiring free facilities (i.e., building and equipment); others believe that only certain services must be provided free of charge. “We have the constitution … but interpretation has been an issue. So far, we have not been able to interpret it.” In addition, “constitutionally speaking, there is a big problem at the [presidential] level. Policymakers and politicians believe that health insurance is something that gets money from people, which is against the law.”

### Quality of health services

Quality of care is an important precondition for introducing health insurance, and health insurance is feasible when potential health insurance beneficiaries can access an acceptable quality of care. Interviewees identified the quality of health services as a key issue for implementing health insurance. In Afghanistan, the population perceives the quality of care to be low. Although the BPHS and Essential Package of Hospital Services (EPHS) are theoretically free of charge, patients must purchase medications from pharmacies and obtain lab tests and examinations from private providers because of medications stockouts and lack of medical equipment and lab tests. They thus end up paying much higher fees: “Patients mostly pay for medicine and diagnostic examinations that are not available in [the] majority of our hospitals.”

The low quality of healthcare services in Afghanistan presents a challenge for the introduction of a health insurance system and affects people’s willingness to participate in an insurance scheme or pay for services at any level.

“My suggestion, before implementing health insurance, is that first and foremost we need to strengthen our health service performance so that our people feel that now that they have insurance, they receive quality health services when they need it.”

Poor quality of services also affects health financing as a whole. For those services not available or of low quality, Afghan citizens leave the country, thereby spending those funds outside of the Afghan economy: “For some procedures and surgeries, like laparoscopy, our people still go to other countries, like India, Pakistan, and Turkey.” Seeking care abroad due to poor quality of services often results in high levels of OOP expenditure.

### Population awareness and trust

Although Afghanistan had experience with health insurance for civil servants in the 1970s and community-based health insurance in five districts in the early 2000s, health insurance, which requires prepayment (premiums) to allow enrollees to receive health care at discounted costs or no costs when seeking care, is a new concept for most of the population. Many people think that paying money for insurance is worthless. The high illiteracy and poverty rates pose further challenges to educating the population about health insurance. Even highly educated people who work in the formal sector, such as NGOs, would prefer to receive a medical allowance from their employer than to join health insurance programs.

“We are planning to start it [health insurance] from Jan, 2015 … But we understand [that] we don’t succeed to educate the educated people to tell them why we want this [health insurance]. Now people, even a person with a PhD degree, tell us no. Because if we give them medical insurance, we will cut the medical allowance … People are not familiar with the basic concept of health insurance. You educate people about the concept; it is a very difficult job according to my experience after one year education.”

Our interviews found that communities lack trust not only regarding Afghan insurance systems but also more generally in the government. “There is reluctance from those stakeholders to contribute to [a] public insurance scheme, because there is no equation of trust of government.” This prevalent lack of trust extends to both the private and public sectors, and relates to the lack of security and volatile economic situation of the country. This general distrust creates an interesting phenomenon: the government feels the public does not trust private health insurance programs, and private companies feel the public does not trust government programs due to prevalent corruption and a sense that the government is unable to oversee a health insurance scheme effectively.

### Fiscal space and willingness and ability to pay

Stakeholders felt that in addition to the government’s low institutional capacity to collect revenues, the country’s large informal economy poses a challenge to revenue generation. The informal working sector also does not allow for effective collection of taxes on small private enterprises.

“Health insurance or life insurance is applicable when people have job[s], and from their income some amount is deducted and transferred to his/her insurance for use when they need. However, in Afghanistan more than 65% of population is jobless.”

“Not all people have a regular salary and [they] don’t know their income. In this country, the income of a farmer is not predictable. It depends on the rain level. It changes from year to year.”

Stakeholders had mixed opinions on the population’s willingness to pay. On the one hand, the demand for health insurance is increasing in the private market. Large organizations working in Afghanistan are actively looking for suitable health insurance plans for their employees, largely because of the insecure working environment. In addition, health is often the second most important issue for the population, after food security. The high poverty rate and low awareness of health insurance limit the population’s willingness to pay. Additionally, the low quality of healthcare further reduces people’s willingness to pay for health insurance: “… quality again is an issue. Everyone would be willing to pay for quality services but [for] bad quality services, no one would be willing to pay; at least I’m not willing to pay.” Various stakeholders also expressed concerns for ensuring protection for the poor should new financing strategies be enacted.

### Capacity to implement and manage a health insurance system

Many stakeholders felt that there was limited technical and managerial capacity to operate a health insurance system in Afghanistan. The number of people dedicated to working on insurance issues is limited to a few select government units, further highlighting the current lack of capacity: “In all of the MoPH, only 1 or 2% [are] working in health insurance.” To date, there has been limited capacity building and training related to health insurance design and management, resulting in a lack of capacity at all levels of the government: “We don’t have the capacity to manage the scheme … and unfortunately there is no capacity even at the central level.” A consensus exists amongst stakeholders that donor support for health insurance should focus on technical assistance and capacity building: “We need a lot of help from donors and government to train us and teach us about insurance topics.”

On the contrary, some stakeholders were optimistic about getting young talent to engage in operating health insurance schemes. Private insurance companies that have begun to provide health insurance products also expressed willingness to support such an operation.

“In the last 13 years, … some of our workers and people in Afghanistan increase their capacity. … In addition, there are many of universities in private and public sector. Many of our young generation graduated from universities, especially in the field of medicine. Graduates from universities can do and run the health insurance program in Afghanistan.”

## Discussion

Almost all of the stakeholders from government agencies, private sector, donors, and UN agencies are interested in introducing health insurance in Afghanistan, and acknowledge the limitations in the current health system to provide adequate healthcare and financial protection to its population. With the expected decline in donor funding over the next ten years, gradually obtaining alternative sources of funding for healthcare and introducing prepayment mechanisms are more imperative than ever; this fact is reflected by the most recent health financing strategy developed by the Afghanistan MoPH [20].

Despite their interest, stakeholders foresee great challenges based on the findings from this study. The major challenges are lack of high-level consistent commitment to health insurance, ambiguity in the constitution, low quality of care, a low level of public awareness of health insurance, limited management capacity to run health insurance, and limited fiscal space and willingness to pay for health insurance. Stakeholders acknowledge that many efforts, discussed in detail in this section, require addressing these challenges to prepare the country for establishing health insurance schemes.

Similar to many health reform initiatives in Afghanistan and other developing countries, establishing health insurance schemes, whether public or private, requires strong political commitment [21–23]. It is paramount to build understanding and consensus among high-level government officials that health insurance should be considered as a means to strengthen the healthcare system for increased sustainability.

The donor community has helped the MoPH initiate efforts toward building political will and HEFD has advocated for risk-pooling mechanisms by approaching the MoPH and government officials from different ministries. These activities have helped the government better understand the benefits and applicability of health insurance in Afghanistan. Political commitment must go beyond the MoPH. Implementing health insurance will involve multiple government agencies. The MoPH will need to collaborate closely with other ministries and agencies to address the barriers to implementing health insurance in the country. In addition, given Afghanistan’s heavy dependence on external aid and technical support, donors and international agencies should use their unique positions in the health system to move health insurance onto the government’s agenda as a mechanism of achieving UHC.

Stakeholders identified Article 52 of the constitution a major legal and regulatory barrier, which stipulates that, “The state is obliged to provide free means of preventive healthcare and medical treatment, and proper health facilities to all citizens of Afghanistan in accordance with the law.” Although the government would like to commit to free care, in reality, health services are not free. Households finance 73% of total health spending. This demonstrates the lack of clarity that exists related to the constitution—which services should be provided free of cost and which should not, and the extent to which the constitution would limit the use of health insurance. High-level government officials hold different opinions on the correct interpretation of the law. The ambiguity of this article has led to a deadlock in further pursuing health insurance and other alternative potential health financing mechanisms.

Recent discussions have begun within the MoPH to draft a revised amendment clarifying the areas of health services that should remain free of charge and those for which payments can be collected. As the interviews showed, there is general agreement that primary healthcare services through the BPHS should remain financially accessible to all. Additional services at the EPHS level beyond the basic package may be considered chargeable, as well as secondary- and tertiary-level healthcare services.

Low quality of healthcare is another major concern that prevents establishing health insurance. In Afghanistan, the low quality of health services has been widely criticized as inadequate to meet the population’s health needs. Only when the quality of services improves will people be confident enough to join health insurance to seek care at contracted health facilities. Although the quality of services has improved substantially in the last decade through the BPHS and EPHS [24], and through the introduction of the Minimum Required Standards (MRS) for private sector hospitals, health services need to be further strengthened and standardized. Among quality issues, shortages of drugs and medical equipment are fundamental. The government already has begun working toward addressing quality issues. Several major initiatives have been taken, which include (1) a program enhancing hospital autonomy; (2) “contracting out” service delivery to NGOs; (3) results-based financing (RBF) pilot program to reward better performance; and (4) the introduction of MRS and a certification process in the private sector.

One important the step that Afghanistan is taking is aiming for accreditation of both public and private health facilities for quality assurance. Initial steps are underway in Afghanistan to establish an accreditation system and an accreditation oversight body. A comprehensive assessment found that that the country is ready for such an initiative and should begin the necessary steps toward establishing a system immediately [25]. Several low- and middle-income countries, including Rwanda and Jordan, have introduced accreditation procedures and generated positive results [26–28], which provide valuable lessons for Afghanistan.

Low public awareness of health insurance also poses substantial challenges for Afghanistan to initiate health insurance. Interviews with private health insurance companies showed an increasing demand for health insurance in the country, particularly among the wealthy population who work for international organizations, banks, and NGOs. However, the concept of health insurance has not been widely discussed and disseminated. More sensitization activities on health insurance among the population is needed to raise awareness in the country about the topic. The MoPH has experience in promoting anti-tobacco and health behavior messages through different means. Although the content of health insurance may be somewhat more complex to communicate, the mechanisms for distributing messages and developing materials are available from the MoPH’s Health Promotion Department.

Initiating health insurance will not be possible without international financial and technical support. The government’s capacity to begin a health insurance scheme remains limited—not only the capacity to design and operate health insurance, but also to increase the fiscal space of the healthcare system and gain trust from the population regarding publicly funded programs.

Initial efforts to enhance health sector capacity for managing health insurance are underway. Private health insurance may be premature; only a few private insurance companies have started health insurance products or acting as brokers and transferring financial risks to reinsurers outside of Afghanistan. However, given the increasing interest in insurance, the private sector is building its capacity to manage health insurance schemes by recruiting young talent and receiving technical support from their overseas reinsurance companies. In the public sector, few government staff are trained on how to operate health insurance schemes; building technical capacities for health insurance early in the process will be critical for the roll out and success of future insurance schemes.

Efforts to improve transparency and to build trust have been taken in the country. Often, the general population in Afghanistan has mixed feelings about public programs for healthcare. The history of ethnic and political conflict and fragile governance in Afghanistan has created a mix of expectations and distrust of the government. On one hand, the population expects the government to take a major role in providing and financing healthcare and, on the other hand, people are concerned about the implementation and effectiveness of such programs and do not trust public programs due to the prevalent corruption. Improving the transparency and accountability of the government to the public would mitigate such concerns. The MoPH has conducted the National Health Accounts (NHA) and Public Expenditure Tracking Survey (PETS), and developed the Expenditure Management Information System (EMIS). These activities contribute to anti-corruption efforts in the country and these efforts and their results should be communicated to the population in an easy-to-understand way.

With limited resources for health, the government’s taxation capacity should be strengthened to increase the fiscal space for health to allow health insurance to be implemented. In fact, revenue collection in Afghanistan has increased significantly since 2002; totaling US$2.04 billion in 2011–12, with an annual increase of 16%, resulting in more than nine times the level of revenue collected in 2002. Revenue as a proportion of GDP was more than 11.6% in 2011–12, up from 11.3% in 2010–11 and 3% in 2002 [4]. However, this number remains lower than the average of 15.2% in low-income countries and of 25.6% in lower mid-income countries in 2011 [29]. The MoPH should coordinate with the MoF and the new USAID-supported tax revenue collection project to gain a better understanding of how taxation could be strengthened and how funds could be allocated to public health services. Additionally, anti-corruption efforts, institutionalization and automation of tax administration processes, and expansion of the number of taxpayers would improve the government’s financial situation and create fiscal space for healthcare, thus alleviating concerns caused by severe budget constraints, such as lack of medical equipment and weak health infrastructure (i.e., poorly maintained buildings).

We acknowledge several limitations of this study. Firstly, the majority of interviews were conducted in English. Despite many Afghans speaking English fluently, the interview language may remain a barrier for communication, leading to the loss of some information. Similarly, for those interviewees who did not speak English, the research team had to rely on the translator to communicate. Secondly, health insurance is quite a technical and complex topic and requires a deep understanding of health systems and a country’s political landscape to provide sensible information for policymaking. Those who participated in the KIIs and FGDs with an understanding of health insurance might not necessarily represent the stakeholder’s opinion as an agency, although the balance between technical and political competence of the participants was considered. Despite these limitations, training for interviewers and the translator, and consultation with the MoPH to select participants, may help reduce potential biases.

## Conclusions

Given the existence of major barriers to health insurance, such as legal restrictions, low quality of care, and low fiscal space, establishment of a health insurance scheme is not expected in the short term (one to two years). However, this circumstance suggests that the government and donors must work together to progressively prepare the country for introducing health insurance. Afghanistan could learn from the experience of some low-income countries in establishing health insurance, such as Rwanda and Ghana [8], where both community-based health insurance and compulsory health insurance in the formal sector are implemented.

The next few years will be critical in reshaping Afghanistan’s healthcare system, as they will provide a limited period of donor-funded BPHS and EPHS services, allowing the government to develop alternative mechanisms to generate domestic resources for healthcare. Afghanistan will need to progressively address the major concerns that impede the establishment of health insurance and take an incremental approach to building a health insurance system.

In the 2012–2020 Health Financing Policy, the MoPH stated its intent to implement UHC in Afghanistan. If Afghanistan is to continue to move toward UHC, it must address the health financing challenges that exist in the system, particularly as donor funding declines over the next decade. Introducing health insurance would be a significant step in moving the country toward UHC and address current challenges in the system. In light of the identified barriers to establishing health insurance in the country, the Afghanistan government and international partners need to work together to address those barriers and accelerate progress toward achieving UHC.

## Additional file

Additional file 1: (DOCX 457 kb)

## Abbreviations

EC: European Commission
EMIS: Expenditure management information system
FGDs: focus group discussions
HEFD: Health Economics and Financing Directorate
ILO: International labor office
KIIs: Key informant interviews
MoH: Ministry of Justice
MoPH: Ministry of Public Health
NGOs: Nongovernmental organizations
NHA: National health accounts
OOP: Out-of-pocket
PETS: Public expenditure tracking survey
RBF: Results-based financing
STEP: Social exclusion and poverty global program
THE: Total health expenditure
UHC: Universal health coverage
USAID: The United States Agency for International Development
WHO: World Health Organization
