# Supplementary material for: Assessing the feasibility of introducing health insurance in Afghanistan: a qualitative stakeholder analysis
Source: BMC Health Serv Res. 2017 Feb 22;17:157. doi: 10.1186/s12913-017-2081-y (PMC5320667; doi:10.1186/s12913-017-2081-y)
Supplement: Additional file 1: — (DOCX 457 kb) [file 12913_2017_2081_MOESM1_ESM.docx]

**Interviewer Information**

| Name: | Date: ___/___/_____ |
| --- | --- |
| Time: | Location: |

1. **Introduction**

The Health Policy Project is conducting a study on behalf of the Ministry of Public Health to examine feasibility of re-introducing health insurance in Afghanistan. Afghanistan experienced health insurance in the 1970s. The health insurance was abolished due to decades-long conflict. Since then, no health insurance schemes have been established. Currently, health services a provided free of charge by NGOs and government facilities under the Basic Package of Health Services and the Essential Package of Hospital Services. Patients also seek care in private clinics and hospitals.

We would like to solicit your opinions on implementing health insurance schemes and what impact such schemes could have on population health outcomes and the health system in Afghanistan. We will ask a list of questions about health insurance, and implementing health insurance in the country. There are no right or wrong answers in this discussion. We are interested in knowing what you think, so please feel free to be frank and to share your point of view, regardless of whether you agree or disagree.

1. **General Information**

- Position
- Length of time in position (years/months)
- Your responsibilities in the parliament

1. **Health System in Afghanistan**

- Are you familiar with the health financing and health delivery situation in the country?
- According to your experience, what are the major challenges for Afghanistan health system to provide quality of health care and protect population from poverty due to illness? How can these challenges or risks be addressed or mitigated by the legal system in the future? *Probe for availability of domestic funds, ability and capacity of government to pay, legal constraints to introducing user fees, etc…*
- From legal perspective, what are the major challenges and opportunities to enhance the health care system? How to overcome challenges?

1. **Regulatory Environment for Health**

- Please describe the regulatory environment for healthcare services
- What laws govern issues such as
  - Paying for services. *Probe for familiarity with health law, and opinion on payment for services.*
  - Quality of care. *Probe for accreditation, quality standards/regulations, private and public sectors.*
  - Regulation of healthcare services

1. **Perceptions of Health Insurance**

- What is your opinion of introducing health insurance in Afghanistan as a way to finance health services? *Probe for perceptions of health insurance, experience, where opinions are coming from, feasibility, why they think it’s feasible or not, why they support or not, etc..*
  - If not support, what are the major reasons for not supporting?
    - *Probe for:*
      - *Concerns on legal framework and political stability and commitment*
      - *Concerns on institutional capacity to manage health insurance schemes (regulation and monitoring) and providing quality services (human resources and infrastructure)*
      - *Concerns on sustainable financing*
      - *Concerns on quality of services*
      - *Concerns on acceptability of population (trust of health insurance agencies and government, ability to pay, access, and culture)*
      - *Other concerns (e.g. security)*
      - *What strategies can address your potential concerns*
    - Do you think the country will ever be ready for health insurance? *Why? If so, when and how? What are some ways to reduce high out of pocket payments by households for health services?*
  - If support, what are the major reasons? *Probe*
    - *The same dimensions mentioned above.*
    - What is the health insurance model that you think is best applicable in Afghanistan? *Probing who will finance (government, household, donors, or combination of them), who should manage the health insurance (independent, semi-independent agency), who should be covered, what are services should be covered, who should provide services. Particular, probe health insurance model for the poor.*
    - What are potential challenges to implement your proposed health insurance? How to overcome them?
    - What is the most important, in order to successfully implement health insurance schemes in Afghanistan?
- In the 1970’s Afghanistan had a social health insurance system. Do you know what the regulations and laws were that guided the health insurance system?
- In Afghanistan, in the past few years some private health insurance companies have been established. Please describe how health insurance schemes are regulated in Afghanistan and what your opinion is of the government’s capacity to currently regulate these schemes.

**Interviewer Information**

| Name: | Date: ___/___/_____ |
| --- | --- |
| Time: | Location: |

1. **Introduction**

The Health Policy Project is conducting a study on behalf of the Ministry of Public Health to examine feasibility of re-introducing health insurance in Afghanistan. Afghanistan experienced health insurance in the 1970s. The health insurance was abolished due to decades-long conflict. Since then, no health insurance schemes have been established. Currently, health services are provided free of charge by NGOs and government facilities under the Basic Package of Health Services and the Essential Package of Hospital Services. Patients also seek care in private clinics and hospitals.

We would like to solicit your opinions on implementing health insurance schemes and what impact such schemes could have on population health outcomes and the health system in Afghanistan. We will ask a list of questions about health insurance, and implementing health insurance in the country. There are no right or wrong answers in this discussion. We are interested in knowing what you think, so please feel free to be frank and to share your point of view, regardless of whether you agree or disagree.

1. **General Information**

*The purpose of this section is to obtain general background information about the respondent, probe for the following:*

- Position
- Length of time in position (years/months)
- Responsibilities of the ministry in Afghanistan and the position overall, and specific to health financing
- The ministry’s priorities in the health sector

1. **Financing health in Afghanistan**

- Are you familiar with how Afghanistan finances its health system?
- What are the major opportunities and challenges to finance health? How can the challenges or risks be addressed or mitigated in the future? *Probe for availability of domestic funds, ability and capacity of government to pay, legal constraints to introducing user fees, etc…*
- Do you know what the current government budget for health is? Is the amount sufficient for health (yes or no)?
  - If yes, please provide justifications
  - If no, do you think MOF will increase budget for health, and how government will collect more resources for health (i.e. strengthen tax collection capacity, more progressive tax, and earmarked tax)?
- From MoF’s perspective, what is the best way to finance health (Government tax financing, social health insurance, out of pocket spending or combination of them)?
- What do you think is the best way to engage MoF in direct dialogue with MoPH on financing health? *Do any mechanisms currently exist? How can they be improved?*
- What are the activities that MOF is doing and planning to improve the financial situation for health?

1. **Perceptions of Health Insurance**

- What is your opinion of introducing health insurance in Afghanistan as a way to finance health services? *Probe for perceptions of health insurance, experience, where opinions are coming from, feasibility, why they think it’s feasible or not, why they support or not, etc..*
  - If not support, what are the major reasons for not supporting?
    - *Probe for:*
      - *Concerns on legal framework and political stability and commitment*
      - *Concerns on institutional capacity to manage health insurance schemes (regulation and monitoring) and providing quality services (human resources and infrastructure)*
      - *Concerns on sustainable financing*
      - *Concerns on quality of services*
      - *Concerns on acceptability of population (trust of health insurance agencies and government, ability to pay, access, and culture)*
      - *Other concerns (e.g. security)*
      - *What strategies can address your potential concerns*
    - Do you think the country will ever be ready for health insurance? *Why? If so, when and how? What are some ways to reduce high out of pocket payments by households for health services?*
  - If support, what are the major reasons? *Probe*
    - *The same dimensions mentioned above.*
    - What is the health insurance model that you think is best applicable in Afghanistan? *Probing who will finance (government, household, donors, or combination of them), who should manage the health insurance (independent, semi-independent agency), who should be covered, what are services should be covered, who should provide services. Particular, probe health insurance model for the poor.*
    - How will MOF support the health insurance schemes?
    - What are potential challenges to implement your proposed health insurance? How to overcome them?
    - What is the most important, in order to successfully implement health insurance schemes in Afghanistan?
- In Afghanistan, in the past few years some private health insurance companies have been established. Which, if any, health insurance companies have you heard about or are familiar with and what are your opinions?
  - Please describe how health insurance schemes are regulated in Afghanistan, and how private health insurance agencies are taxed.
  - Are there any lessons learnt from this current experience with private health insurance?

**Interviewer Information**

| Name: | Date: ___/___/_____ |
| --- | --- |
| Time: | Location: |

**Introduction**

The Health Policy Project is conducting a study on behalf of the Ministry of Public Health to examine feasibility of re-introducing health insurance in Afghanistan. Afghanistan experienced health insurance in the 1970s. The health insurance was abolished due to decades-long conflict. Since then, no health insurance schemes have been established. Currently, health services a provided free of charge by NGOs and government facilities under the Basic Package of Health Services and the Essential Package of Hospital Services. Patients also seek care in private clinics and hospitals.

We would like to solicit your opinions on implementing health insurance schemes and what impact such schemes could have on population health outcomes and the health system in Afghanistan. We will ask a list of questions about health insurance, and implementing health insurance in the country. There are no right or wrong answers in this discussion. We are interested in knowing what you think, so please feel free to be frank and to share your point of view, regardless of whether you agree or disagree.

1. **General Information**

- Position:________________
- Length of time in position (years/months):_________________
- Responsibilities of the ministry in Afghanistan and the position
- The ministry’s priorities in the health sector

1. **Health Financing and health service delivery in Afghanistan**

- Are you familiar with the health financing situation and health service delivery in the country?
- What are the major opportunities and challenges to provide health services to population? How can these challenges or risks be addressed or mitigated in the future? *Probe for availability of domestic funds, ability and capacity of government to pay, legal constraints to introducing user fees, etc…*

1. **Perceptions of health insurance**

- What is your opinion of introducing health insurance in Afghanistan? *Probe for perceptions of health insurance, experience, where opinions are coming from, feasibility, why they think it’s feasible or not, why they support or not, etc..*
  - If not support, what are the major reasons for not supporting?
    - *Probe for:*
      - *Concerns on legal framework and political stability and commitment*
      - *Concerns on institutional capacity to manage health insurance schemes (regulation and monitoring) and providing quality services (human resources and infrastructure)*
      - *Concerns on sustainable financing*
      - *Concerns on quality of services*
      - *Concerns on acceptability of population (trust of health insurance agencies and government, ability to pay, access, and culture)*
      - *Other concerns (e.g. security)*
      - *What strategies can address your potential concerns*
    - Do you think the country will ever be ready for health insurance? *Why? If so, when and how? What are some ways to reduce high out of pocket payments by households for health services?*
  - If support, what are the major reasons? *Probe*
    - *The same dimensions mentioned above.*
    - What is the health insurance model that you think is best applicable in Afghanistan? *Probing who will finance (government, household, donors, or combination of them), who should manage the health insurance (independent, semi-independent agency), who should be covered, what are services should be covered, who should provide services. Particular, probe health insurance model for the poor.*
    - What are potential challenges to implement your proposed health insurance? How to overcome them?
    - What is the most important from the MoPH’s perspective, in order to successfully implement health insurance schemes in Afghanistan?
- In Afghanistan, in the past few years some private health insurance companies have been established. Which, if any, health insurance companies have you heard about or are familiar with and what are your opinions?
  - Please describe current government policy about health insurance.
  - Are there any lessons learnt from this current experience with private health insurance?

1. **Health care reform**

- What are the health care reform activities that are ongoing and planned?
- In your opinion, what is the most imperative reform that should be done for providing health services and protect households from financial risks?
- What is your opinion of ongoing reform activities? What are the successes and challenges?
- How do you envision the establishment of health insurance to be integrated into ongoing health care reform activities?

**Interviewer Information**

| Name: | Date: ___/___/_____ |
| --- | --- |
| Time: | Location: |

1. **Introduction**

The Health Policy Project is conducting a study on behalf of the Ministry of Public Health to examine feasibility of re-introducing health insurance in Afghanistan. Afghanistan experienced health insurance in the 1970s. The health insurance was abolished due to decades-long conflict. Since then, no health insurance schemes have been established. Currently, health services a provided free of charge by NGOs and government facilities under the Basic Package of Health Services and the Essential Package of Hospital Services. Patients also seek care in private clinics and hospitals.

We would like to solicit your opinions on implementing health insurance schemes and what impact such schemes could have on population health outcomes and the health system in Afghanistan. We will ask a list of questions about health insurance, and implementing health insurance in the country. There are no right or wrong answers in this discussion. We are interested in knowing what you think, so please feel free to be frank and to share your point of view, regardless of whether you agree or disagree.

1. **General Information**

- Position
- Length of time in position (years/months)
- Responsibilities of the ministry in Afghanistan and the position overall, and specific to health financing
- The ministry’s priorities in the health sector

1. **Health System in Afghanistan**

- Are you familiar with the health financing and health delivery situation in the country?
- According to your experience, what are the major challenges for Afghanistan health system to provide quality of health care and protect population from poverty due to illness? How can these challenges or risks be addressed or mitigated by the legal system in the future? *Probe for availability of domestic funds, ability and capacity of government to pay, legal constraints to introducing user fees, etc…*
- From legal perspective, what are the major challenges and opportunities to enhance the health care system? How to overcome challenges?

1. **Regulatory Environment for Health**

- Please describe the regulatory environment for healthcare services
- What laws govern issues such as
  - Paying for services. *Probe for familiarity with health law, and opinion on payment for services.*
  - Quality of care. *Probe for accreditation, quality standards/regulations, private and public sectors.*
  - Regulation of healthcare services

1. **Perceptions of Health Insurance**

- What is your opinion of introducing health insurance in Afghanistan as a way to finance health services? *Probe for perceptions of health insurance, experience, where opinions are coming from, feasibility, why they think it’s feasible or not, why they support or not, etc..*
  - If not support, what are the major reasons for not supporting?
    - *Probe for:*
      - *Concerns on legal framework and political stability and commitment*
      - *Concerns on institutional capacity to manage health insurance schemes (regulation and monitoring) and providing quality services (human resources and infrastructure)*
      - *Concerns on sustainable financing*
      - *Concerns on quality of services*
      - *Concerns on acceptability of population (trust of health insurance agencies and government, ability to pay, access, and culture)*
      - *Other concerns (e.g. security)*
      - *What strategies can address your potential concerns*
    - Do you think the country will ever be ready for health insurance? *Why? If so, when and how? What are some ways to reduce high out of pocket payments by households for health services?*
  - If support, what are the major reasons? *Probe*
    - *The same dimensions mentioned above.*
    - What is the health insurance model that you think is best applicable in Afghanistan? *Probing who will finance (government, household, donors, or combination of them), who should manage the health insurance (independent, semi-independent agency), who should be covered, what are services should be covered, who should provide services. Particular, probe health insurance model for the poor.*
    - What are potential challenges to implement your proposed health insurance? How to overcome them?
    - What is the most important, in order to successfully implement health insurance schemes in Afghanistan?
- In the 1970’s Afghanistan had a social health insurance system. Do you know what the regulations and laws were that guided the health insurance system?
- In Afghanistan, in the past few years some private health insurance companies have been established. Please describe how health insurance schemes are regulated in Afghanistan and what your opinion is of the government’s capacity to currently regulate these schemes.

**Interviewer Information**

| Name: | Date: ___/___/_____ |
| --- | --- |
| Time: | Location: |

**Introduction**

The Health Policy Project is conducting a study on behalf of the Ministry of Public Health to examine feasibility of re-introducing health insurance in Afghanistan. Afghanistan experienced health insurance in the 1970s. The health insurance was abolished due to decades-long conflict. Since then, no health insurance schemes have been established. Currently, health services a provided free of charge by NGOs and government facilities under the Basic Package of Health Services and the Essential Package of Hospital Services. Patients also seek care in private clinics and hospitals.

We would like to solicit your opinions on implementing health insurance schemes and what impact such schemes could have on population health outcomes and the health system in Afghanistan. We will ask a list of questions about health insurance, and implementing health insurance in the country. There are no right or wrong answers in this discussion. We are interested in knowing what you think, so please feel free to be frank and to share your point of view, regardless of whether you agree or disagree.

1. **General Information**

- Position:________________
- Length of time in position (years/months):_________________
- Responsibilities of the ministry in Afghanistan and the position
- The ministry’s priorities in the health sector

1. **Health Financing and health service delivery in Afghanistan**

- Are you familiar with the health financing situation and health service delivery in the country?
- What are the major opportunities and challenges to provide health services to population? How can these challenges or risks be addressed or mitigated in the future? *Probe for availability of domestic funds, ability and capacity of government to pay, legal constraints to introducing user fees, etc…*

1. **Perceptions of health insurance**

- What is your opinion of introducing health insurance in Afghanistan? *Probe for perceptions of health insurance, experience, where opinions are coming from, feasibility, why they think it’s feasible or not, why they support or not, etc..*
  - If not support, what are the major reasons for not supporting?
    - *Probe for:*
      - *Concerns on legal framework and political stability and commitment*
      - *Concerns on institutional capacity to manage health insurance schemes (regulation and monitoring) and providing quality services (human resources and infrastructure)*
      - *Concerns on sustainable financing*
      - *Concerns on quality of services*
      - *Concerns on acceptability of population (trust of health insurance agencies and government, ability to pay, access, and culture)*
      - *Other concerns (e.g. security)*
      - *What strategies can address your potential concerns*
    - Do you think the country will ever be ready for health insurance? *Why? If so, when and how? What are some ways to reduce high out of pocket payments by households for health services?*
  - If support, what are the major reasons? *Probe*
    - *The same dimensions mentioned above.*
    - What is the health insurance model that you think is best applicable in Afghanistan? *Probing who will finance (government, household, donors, or combination of them), who should manage the health insurance (independent, semi-independent agency), who should be covered, what are services should be covered, who should provide services. Particular, probe health insurance model for the poor.*
    - What are potential challenges to implement your proposed health insurance? How to overcome them?
    - What is the most important from the MoL’s perspective, in order to successfully implement health insurance schemes in Afghanistan?
- In Afghanistan, in the past few years some private health insurance companies have been established. Which, if any, health insurance companies have you heard about or are familiar with and what are your opinions?
  - Please describe current government policy about health insurance.
  - Are there any lessons learnt from this current experience with private health insurance?

1. **Health care reform**

- What are the health care reform activities that are ongoing and planned?
- In your opinion, what is the most imperative reform that should be done for providing health services and protect households from financial risks?
- What is your opinion of ongoing reform activities? What are the successes and challenges?
- How do you envision the establishment of health insurance to be integrated into ongoing health care reform activities?

**Interviewer Information**

| Name: | Date: ___/___/_____ |
| --- | --- |
| Time: | Location: |

**Introduction**

The Health Policy Project is conducting a study on behalf of the Ministry of Public Health to examine feasibility of re-introducing health insurance in Afghanistan. Afghanistan experienced health insurance in the 1970s. The health insurance was abolished due to decades-long conflict. Since then, no health insurance schemes have been established. Currently, health services a provided free of charge by NGOs and government facilities under the Basic Package of Health Services and the Essential Package of Hospital Services. Patients also seek care in private clinics and hospitals.

We would like to solicit your opinions on implementing health insurance schemes and what impact such schemes could have on population health outcomes and the health system in Afghanistan. We will ask a list of questions about health insurance, and implementing health insurance in the country. There are no right or wrong answers in this discussion. We are interested in knowing what you think, so please feel free to be frank and to share your point of view, regardless of whether you agree or disagree.

1. **General Information**

- Position:________________
- Length of time in position (years/months):_________________

1. **General Information of the Insurance Agency**

- When was the insurance agencies established___________
- Number of employees_____________________________
- How is the insurance agency structured?
- Number of enrolees ______________________________

1. **Health Insurance designed**

- Who are the enrolees (i.e. in group or individual)? *Probe socio-economic, occupation, geography, age, and gender variation; time trend of coverage, and turnover of enrolees.*
- What benefits are included in the service package (i.e. primary care vs secondary and tertiary care), and what are the basis for the design of the benefit.
- Are there any deductibles, coinsurance or copayment and reimbursement ceiling?
- What is the premium of the insurance? How is it determined and calculated? How do you collect the premium?
- Who are the health providers for your agency? How do you select them? How do you contract providers and pay them (for outpatient, inpatient, and emergency services).
- How do you manage medical claims and monitor fraudulence?
- How is the insurance marked? What is your prediction of your future market?
- How is the financial situation of your agency in the last five years

1. **Regulation of health insurance agency**

- To whom were you registered? Are there regulations to register a health insurance agency?
- What are the major regulations your company need to be aware of
- To further promote health insurance, what suggestions do you have regarding government regulation of health insurance?

1. **Perceptions of health insurance**

- What is your opinion of introducing health insurance in Afghanistan? *Probe for perceptions of health insurance, experience, where opinions are coming from, feasibility, why they think it’s feasible or not, why they support or not, etc.*
  - If not support, what are the major reasons for not supporting?
    - *Probe for:*
      - *Concerns on legal framework and political stability and commitment*
      - *Concerns on institutional capacity to manage health insurance schemes (regulation and monitoring) and providing quality services (human resources and infrastructure)*
      - *Concerns on sustainable financing*
      - *Concerns on quality of services*
      - *Concerns on acceptability of population (trust of health insurance agencies and government, ability to pay, access, and culture)*
      - *Other concerns (e.g. security)*
      - *What strategies can address your potential concerns*
    - Do you think the country will ever be ready for health insurance? *Why? If so, when and how? What are some ways to reduce high out of pocket payments by households for health services?*
  - If support, what are the major reasons? *Probe*
    - *The same dimensions mentioned above.*
    - What is the health insurance model that you think is best applicable in Afghanistan? *Probing who will finance (government, household, donors, or combination of them), who should manage the health insurance (independent, semi-independent agency), who should be covered, what are services should be covered, who should provide services. Particular, probe health insurance model for the poor.*
    - What are potential challenges to implement your proposed health insurance? How to overcome them?
    - What is the most important, in order to successfully implement health insurance schemes in Afghanistan?
- What are the lessons and experience of implementing a health insurance schemes?

**Interviewer Information**

| Name: | Date: ___/___/_____ |
| --- | --- |
| Time: | Location: |

1. **Introduction**

The Health Policy Project is conducting a study on behalf of the Ministry of Public Health to examine feasibility of re-introducing health insurance in Afghanistan. Afghanistan experienced health insurance in the 1970s. The health insurance was abolished due to decades-long conflict. Since then, no health insurance schemes have been established. Currently, health services a provided free of charge by NGOs and government facilities under the Basic Package of Health Services and the Essential Package of Hospital Services. Patients also seek care in private clinics and hospitals.

We would like to solicit your opinions on implementing health insurance schemes and what impact such schemes could have on population health outcomes and the health system in Afghanistan. We will ask a list of questions about health insurance, and implementing health insurance in the country. There are no right or wrong answers in this discussion. We are interested in knowing what you think, so please feel free to be frank and to share your point of view, regardless of whether you agree or disagree.

1. **General Information**

| SN | Position | Duration |
| --- | --- | --- |
| 1 |  | \|__\|__\| years; \|__\|__\| months |
| 2 |  | \|__\|__\| years; \|__\|__\| months |
| 3 |  | \|__\|__\| years; \|__\|__\| months |
| 4 |  | \|__\|__\| years; \|__\|__\| months |
| 5 |  | \|__\|__\| years; \|__\|__\| months |
| 6 |  | \|__\|__\| years; \|__\|__\| months |
| 7 |  | \|__\|__\| years; \|__\|__\| months |
| 8 |  | \|__\|__\| years; \|__\|__\| months |

1. **Government Support for Health Insurance**

- What is the government’s position on health insurance? *Probe for leadership in different ministries; Presidency; Parliament; and how the new government may or may not change positions*
- What kind of financial commitment can the government provide for health insurance?

1. **Regulatory and Coordination Capacity**

- What is your opinion of the regulatory capacity of the government for health insurance?
- What is your opinion of the institutional and coordination capacity of the government for health insurance? What would be the institutions that are responsible for regulating, implementing, monitoring health insurance? What would the coordinating body be?

1. **Opinion on Health Insurance, Design, Challenges, and Opportunities**

- These are two frameworks for health financing developed by the MoPH and the World Bank for the BPHS and hospitals. Does this framework still reflect the system the MoPH envisions? (You do not have to agree with the framework, but it helps researchers understand your inclination, please provides your rationales)


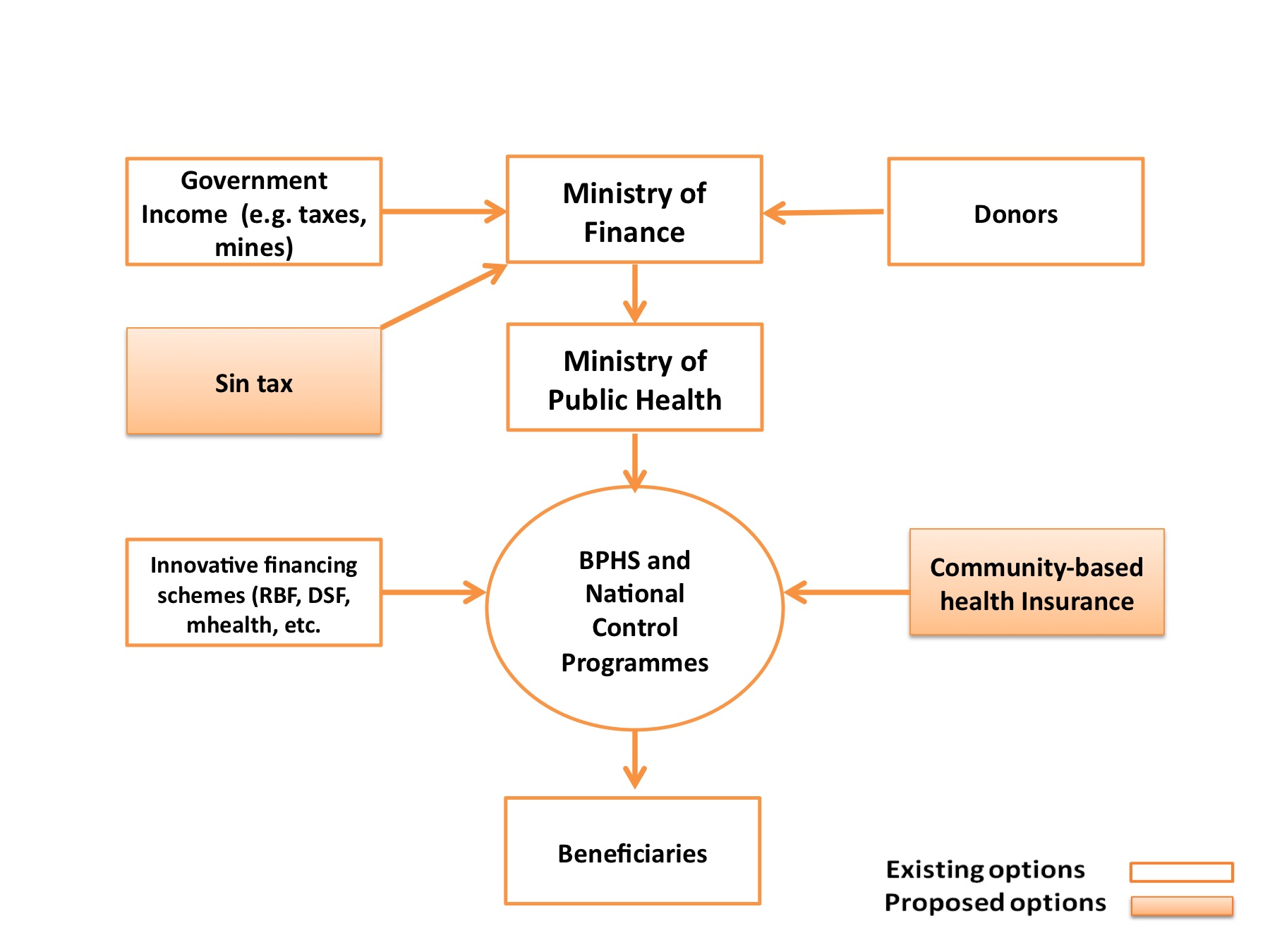


*
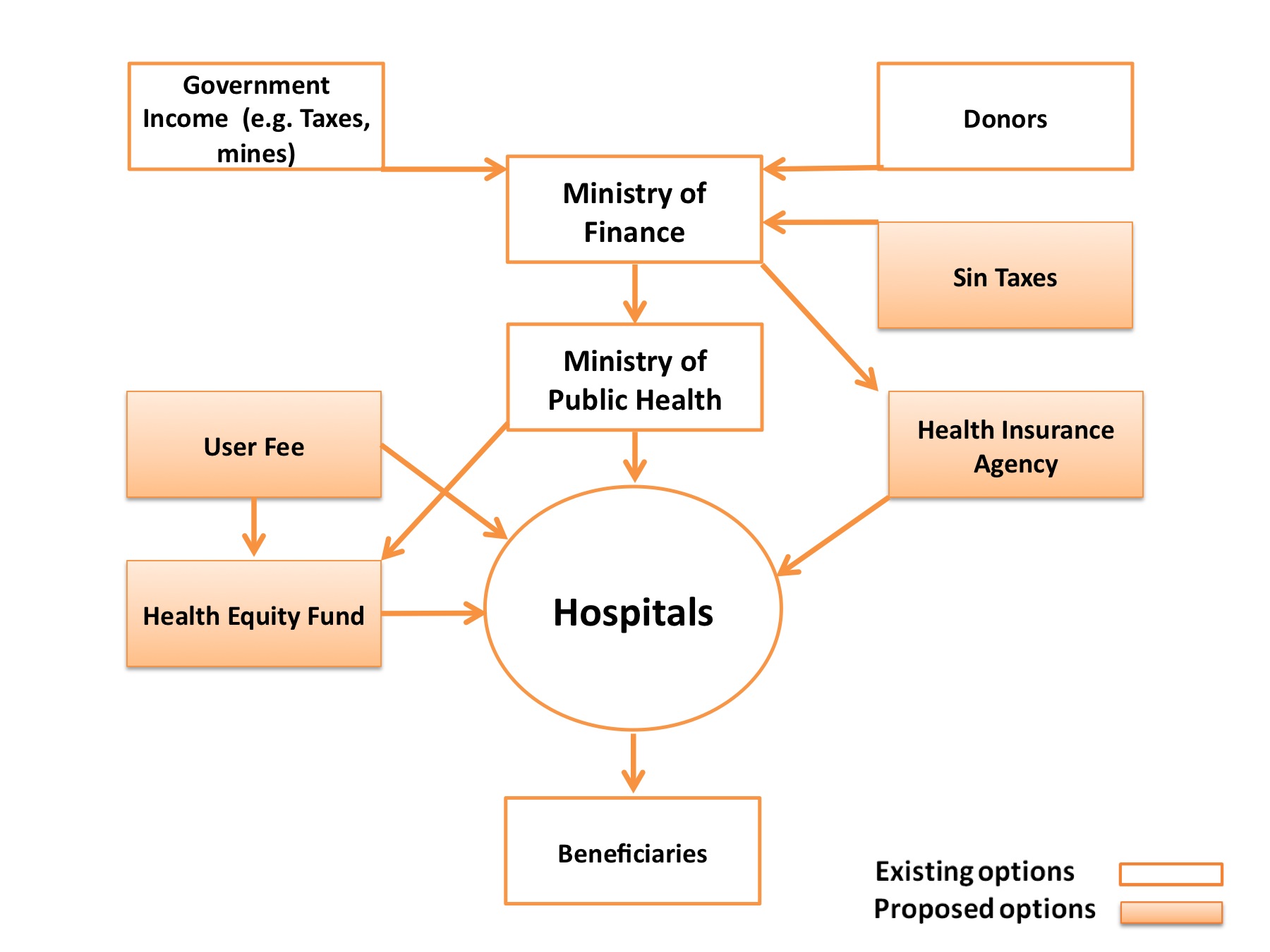
*

- Based on your knowledge and experience, what health insurance schemes do you think are appropriate for Afghanistan? Probe for the following:
  - Beneficiaries:
    - Individual
    - Family
    - Household
    - Employee
  - Geographical coverage
    - Rural
    - Urban
  - Enrollment mechanisms
    - Voluntary
    - Mandatory
  - Administrators: *who should manage the health insurance body? (MoPH, MoF, independent/semi-independent insurance body, etc)*
  - Health services covered under health insurance
  - Cost of health insurance
  - Regulatory body
- Given this description of the health insurance schemes you’ve identified as being feasible for Afghanistan, I’d like to go through a SWOT analysis of introducing health insurance in this country.

|  | **Helpful**  to achieving the objective | **Harmful**  to achieving the objective |
| --- | --- | --- |
| **Internal** origin (attributes of the organization) | STRENGTHS | WEAKNESSES |
| **External** origin (attributes of the environment) | OPPORTUNITIES | THREATS |

**Interviewer Information**

| Name: | Date: ___/___/_____ |
| --- | --- |
| Time: | Location: |

1. **Introduction**

The Health Policy Project is conducting a study on behalf of the Ministry of Public Health to examine feasibility of re-introducing health insurance in Afghanistan. Afghanistan experienced health insurance in the 1970s. The health insurance was abolished due to decades-long conflict. Since then, no health insurance schemes have been established. Currently, health services are provided free of charge by NGOs and government facilities under the Basic Package of Health Services and the Essential Package of Hospital Services. Patients also seek care in private clinics and hospitals.

We would like to solicit your opinions on implementing health insurance schemes and what impact such schemes could have on population health outcomes and the health system in Afghanistan. We will ask a list of questions about health insurance, and implementing health insurance in the country. There are no right or wrong answers in this discussion. We are interested in knowing what you think, so please feel free to be frank and to share your point of view, regardless of whether you agree or disagree.

1. **General Information**

*The purpose of this section is to obtain general background information about the respondents:*

| SN | Position *(note: should all be managers or directors)* | Duration |
| --- | --- | --- |
| 1 |  | \|__\|__\| years; \|__\|__\| months |
| 2 |  | \|__\|__\| years; \|__\|__\| months |
| 3 |  | \|__\|__\| years; \|__\|__\| months |
| 4 |  | \|__\|__\| years; \|__\|__\| months |
| 5 |  | \|__\|__\| years; \|__\|__\| months |
| 6 |  | \|__\|__\| years; \|__\|__\| months |
| 7 |  | \|__\|__\| years; \|__\|__\| months |
| 8 |  | \|__\|__\| years; \|__\|__\| months |

1. **Service Delivery Situation**
   - How is your health facility currently financed and paid
   - *Probe – the source of revenue (government funding, user fees, direct donations, ect..); how does the government pay to the health facilities; financial situation of health facilities; how is the revenue spent for.*
   - Please explain people’s access to health care services provided by public hospitals?
   - What is your perception of the quality of health care services provided by public hospitals??
   - How do people pay for health care services in Afghanistan?
   - *Probe – in Kabul and other urban areas, rural areas; the poor; those employed in the formal sector vs informal sector; monetary or goods/services?; borrow money from relatives or sell land?; estimate of how much households are burdened by costs of health care (clarify that these are in-country costs)*

- What are the major challenges and opportunities for service delivery?
  - *Probe – Human resources, procurement of drugs and equipment, autonomy, financial resources, quality of care, absenteeism, and service coordination.*

1. **Opinions on Health Insurance**

- Are you familiar with health insurance? Can you explain what you think health insurance is? *Probe for any experiences with health insurance (in Afghanistan, out of Afghanistan, 1970’s)*
- What is your opinion of introducing health insurance in Afghanistan as a way to finance health services? *Probe for perceptions of health insurance, experience, where opinions are coming from, feasibility, why they think it’s feasible or not, why they support or not, etc..*
  - If not support, what are the major reasons for not supporting?
    - *Probe for:*
      - *Concerns on legal framework and political stability and commitment*
      - *Concerns on institutional capacity to manage health insurance schemes (regulation and monitoring) and providing quality services (human resources and infrastructure)*
      - *Concerns on sustainable financing*
      - *Concerns on quality of services*
      - *Concerns on acceptability of population (trust of health insurance agencies and government, ability to pay, access, and culture)*
      - *Other concerns (e.g. security)*
      - *What strategies can address your potential concerns*
    - Do you think the country will ever be ready for health insurance? *Why? If so, when and how? What are some ways to reduce high out of pocket payments by households for health services?*
  - If support, what are the major reasons? *Probe*
    - *The same dimensions mentioned above.*
    - What is the health insurance model that you think best applicable in Afghanistan?
      - *Probe who will finance (government, household, donors, or combination of them), who should manage the health insurance, who should be covered, what are services should be covered, who should provide services. Particular, probe health insurance model for the poor.*
    - What are potential challenges to implement your proposed health insurance? How to overcome them?
    - What is most important from a hospital perspective, in order to successfully implement health insurance schemes in Afghanistan?
- In Afghanistan, in the past few years some private health insurance companies have been established. Which, if any, health insurance companies have you heard about or are familiar with and what are your opinions?
- Once health insurance is established, your health facility will be likely to be invited in service provision network and contract with health insurance agencies (either government entity or private entity). Will you be willing to join the service provision network? How would you like to be paid from the health insurance agencies (outpatient services and inpatient services).
- How do you think health insurance will affect the delivery of health services?
  - *Probe for perceived positive impact, negative impact, why? Ask for specific examples of how the service delivery setting might change*

**Interviewer Information**

| Name: | Date: ___/___/_____ |
| --- | --- |
| Time: | Location: |

1. **Introduction**

The Health Policy Project is conducting a study on behalf of the Ministry of Public Health to examine feasibility of re-introducing health insurance in Afghanistan. Afghanistan experienced health insurance in the 1970s. The health insurance was abolished due to decades-long conflict. Since then, no health insurance schemes have been established. Currently, health services are provided free of charge by NGOs and government facilities under the Basic Package of Health Services and the Essential Package of Hospital Services. Patients also seek care in private clinics and hospitals.

We would like to solicit your opinions on implementing health insurance schemes and what impact such schemes could have on population health outcomes and the health system in Afghanistan. We will ask a list of questions about health insurance, and implementing health insurance in the country. There are no right or wrong answers in this discussion. We are interested in knowing what you think, so please feel free to be frank and to share your point of view, regardless of whether you agree or disagree.

1. **General Information**

*The purpose of this section is to obtain general background information about the respondents:*

| SN | Position *(note: should all be managers or directors)* | Duration |
| --- | --- | --- |
| 1 |  | \|__\|__\| years; \|__\|__\| months |
| 2 |  | \|__\|__\| years; \|__\|__\| months |
| 3 |  | \|__\|__\| years; \|__\|__\| months |
| 4 |  | \|__\|__\| years; \|__\|__\| months |
| 5 |  | \|__\|__\| years; \|__\|__\| months |
| 6 |  | \|__\|__\| years; \|__\|__\| months |
| 7 |  | \|__\|__\| years; \|__\|__\| months |
| 8 |  | \|__\|__\| years; \|__\|__\| months |

1. **Service Delivery Situation**
   - How is your health facility currently financed and paid
   - *Probe – the source of revenue (government funding, user fees, direct donations, etc..); existence of funding from the government to the health facilities and in what mechanism; financial situation of health facility; how is the revenue spent for.*
   - Please explain people’s access to health care services provided by private hospitals?
   - *Probe - who use private hospitals, why do they use private hospitals, market share of private providers, and role of private provider in health delivery systems.*
   - What is your perception of the quality of health care services provided by your hospitals? *Probe for challenges*
   - How do your patients pay for health care services in your hospital?
   - *Probe – in Kabul and other urban areas, rural areas; the poor; those employed in the formal sector vs informal sector; monetary or goods/services?; borrow money from relatives or sell land?; estimate of how much households are burdened by costs of health care (clarify that these are in-country costs)*

- What are the major challenges and opportunities for service delivery?
  - *Probe – Human resources, procurement of drugs and equipment, autonomy, financial resources, quality of care, absenteeism, and service coordination.*

1. **Opinions on Health Insurance**

- Are you familiar with health insurance? Can you explain what you think health insurance is?
- In Afghanistan, in the past few years some private health insurance companies have been established. Which, if any, health insurance companies have you heard about or are familiar with and what are your opinions?
  - Does your hospital currently contract with any health insurance agencies to provide health care for its enrollees? Or does your hospital currently provide some form of health insurance to patients?
    - If yes, what is the name of agency, what services are covered, how is your hospital paid.
    - If no, will you hospital be willing to join service provider network to contract with health insurance agency to provide services once health insurance schemes are established.
- What is the private health sector’s experience with health insurance in Afghanistan? *Probe for how these are currently regulated; size; benefits packages; premiums; etc…*
- What is your opinion of introducing health insurance in Afghanistan as a way to finance health services? *Probe for perceptions of health insurance, experience, where opinions are coming from, feasibility, why they think it’s feasible or not, why they support or not, etc..*
  - If not support, what are the major reasons for not supporting?
    - *Probe for:*
      - *Concerns on legal framework and political stability and commitment*
      - *Concerns on institutional capacity to manage health insurance schemes (regulation and monitoring) and providing quality services (human resources and infrastructure)*
      - *Concerns on sustainable financing*
      - *Concerns on quality of services*
      - *Concerns on acceptability of population (trust of health insurance agencies and government, ability to pay, access, and culture)*
      - *Other concerns (e.g. security)*
      - *What strategies can address your potential concerns*
    - Do you think the country will ever be ready for health insurance? *Why? If so, when and how? What are some ways to reduce high out of pocket payments by households for health services?*
  - If support, what are the major reasons? *Probe*
    - *The same dimensions mentioned above.*
    - What is the health insurance model that you think is best applicable in Afghanistan? Probing who will finance (government, household, donors, or combination of them), who should manage the health insurance, who should be covered, what are services should be covered, who should provide services. Particular, probe health insurance model for the poor.
    - What are potential challenges to implement your proposed health insurance? How to overcome them?
    - What is most important from a hospital perspective, in order to successfully implement health insurance schemes in Afghanistan?
- Once health insurance is established, how likely is your health facility to join a service provision network and contract with health insurance agencies (either government entity or private entity). Will you be willing to join the service provision network? How would you like to be paid from the health insurance agencies (outpatient services and inpatient services).
- How do you think health insurance will affect the delivery of health services?
  - *Probe for perceived positive impact, negative impact, why?, ask for specific examples of how the service delivery setting might change*

**Interviewer Information**

| Name: | Date: ___/___/_____ |
| --- | --- |
| Time: | Location: |

1. **Introduction**

The Health Policy Project is conducting a study on behalf of the Ministry of Public Health to examine feasibility of re-introducing health insurance in Afghanistan. Afghanistan experienced health insurance in the 1970s. The health insurance was abolished due to decades-long conflict. Since then, no health insurance schemes have been established. Currently, health services are provided free of charge by NGOs and government facilities under the Basic Package of Health Services and the Essential Package of Hospital Services. Patients also seek care in private clinics and hospitals.

We would like to solicit your opinions on implementing health insurance schemes and what impact such schemes could have on population health outcomes and the health system in Afghanistan. We will ask a list of questions about health insurance, and implementing health insurance in the country. There are no right or wrong answers in this discussion. We are interested in knowing what you think, so please feel free to be frank and to share your point of view, regardless of whether you agree or disagree.

1. **General Information**

*The purpose of this section is to obtain general background information about the respondents:*

| SN | Position *(note: should all be managers or directors)* | Duration |
| --- | --- | --- |
| 1 |  | \|__\|__\| years; \|__\|__\| months |
| 2 |  | \|__\|__\| years; \|__\|__\| months |
| 3 |  | \|__\|__\| years; \|__\|__\| months |
| 4 |  | \|__\|__\| years; \|__\|__\| months |
| 5 |  | \|__\|__\| years; \|__\|__\| months |
| 6 |  | \|__\|__\| years; \|__\|__\| months |
| 7 |  | \|__\|__\| years; \|__\|__\| months |
| 8 |  | \|__\|__\| years; \|__\|__\| months |

1. **Service Delivery Situation**
   - How is your health facility currently financed and paid
   - *Probe – the source of revenue (government funding, user fees, direct donations, ect..); how does the government pay to the health facilities; financial situation of health facilities; how is the revenue spent for.*
   - Please explain people’s access to health care services provided by public clinics?
   - What is your perception of the quality of health care services provided by public clinics?
   - How do people pay for health care services in Afghanistan?
   - *Probe – in Kabul and other urban areas, rural areas; the poor; those employed in the formal sector vs informal sector; monetary or goods/services?; borrow money from relatives or sell land?; estimate of how much households are burdened by costs of health care (clarify that these are in-country costs)*

- What are the major challenges and opportunities for service delivery?
  - *Probe – Human resources, procurement of drugs and equipment, autonomy, financial resources, quality of care, absenteeism, and service coordination.*

1. **Opinions on Health Insurance**

- Are you familiar with health insurance? Can you explain what you think health insurance is? *Probe for any experiences with health insurance (in Afghanistan, out of Afghanistan, 1970’s)*
- What is your opinion of introducing health insurance in Afghanistan? *Probe for perceptions of health insurance, experience, where opinions are coming from, feasibility, why they think it’s feasible or not, why they support or not, etc..*
  - If not support, what are the major reasons for not supporting?
    - *Probe for:*
      - *Concerns on legal framework and political stability and commitment*
      - *Concerns on institutional capacity to manage health insurance schemes (regulation and monitoring) and providing quality services (human resources and infrastructure)*
      - *Concerns on sustainable financing*
      - *Concerns on quality of services*
      - *Concerns on acceptability of population (trust of health insurance agencies and government, ability to pay, access, and culture)*
      - *Other concerns (e.g. security)*
      - *What strategies can address your potential concerns*
    - Do you think the country will ever be ready for health insurance? *Why? If so, when and how? What are some ways to reduce high out of pocket payments by households for health services?*
  - If support, what are the major reasons? Probe
    - *The same dimensions mentioned above.*
    - What is the health insurance model that you think best applicable in Afghanistan?
      - *Probe who will finance (government, household, donors, or combination of them), who should manage the health insurance, who should be covered, what are services should be covered, who should provide services. Particular, probe health insurance model for the poor.*
    - What are potential challenges to implement your proposed health insurance? How to overcome them?
    - What is most important from a primary care perspective, in order to successfully implement health insurance schemes in Afghanistan?
  - In Afghanistan, in the past few years some private health insurance companies have been established. Which, if any, health insurance companies have you heard about or are familiar with and what are your opinions?
- Once health insurance is established, your health facility will be likely to be invited in service provision network and contract with health insurance agencies (either government entity or private entity). Will you be willing to join the service provision network? How would you like to be paid from the health insurance agencies (outpatient services and inpatient services).
- How do you think health insurance will affect the delivery of health services?
  - *Probe for perceived positive impact, negative impact, why? Ask for specific examples of how the service delivery setting might change*

**Interviewer Information**

| Name: | Date: ___/___/_____ |
| --- | --- |
| Time: | Location: |

**Introduction**

The Health Policy Project is conducting a study on behalf of the Ministry of Public Health to examine feasibility of re-introducing health insurance in Afghanistan. Afghanistan experienced health insurance in the 1970s. The health insurance was abolished due to decades-long conflict. Since then, no health insurance schemes have been established. Currently, health services a provided free of charge by NGOs and government facilities under the Basic Package of Health Services and the Essential Package of Hospital Services. Patients also seek care in private clinics and hospitals.

We would like to solicit your opinions on implementing health insurance schemes and what impact such schemes could have on population health outcomes and the health system in Afghanistan. We will ask a list of questions about health insurance, and implementing health insurance in the country. There are no right or wrong answers in this discussion. We are interested in knowing what you think, so please feel free to be frank and to share your point of view, regardless of whether you agree or disagree.

1. **General Information**

- Position:________________
- Length of time in position (years/months):_________________
- Descript the community saving group program (*size, population, process, length, and outcome*)

1. **Service delivery**
   - In the community that you are serving, where do households seek care (public vs. private)
   - *Probe – how far is the health facility; for what services, why do patients seek care in the public or private health facility, how do they pay for the services*
   - What is your perception of the quality of health care services – private vs. public.
   - Outpatient services
   - Children’s health care
   - Maternity care
   - Inpatient hospitals services
   - Overall clinics
   - Overall National Hospitals and EPHS Hospitals compared to private hospitals
   - *Probe for challenges to quality and access*
2. **Health Insurance and Willingness to Pay**

- Are you familiar with health insurance? Can you explain what you think health insurance is?
- Have you or anyone you know ever participated in a health insurance scheme?
- What is your opinion of introducing health insurance in Afghanistan as a way to finance health services? *Probe for perceptions of health insurance, experience, where opinions are coming from, feasibility, why they think it’s feasible or not, why they support or not, etc..*
  - If not support, what are the major reasons for not supporting?
    - Probe for:
    - *Concerns on political stability and commitment*
    - *Concerns on institutional capacity to manage health insurance schemes (regulation and monitoring) and providing quality services (human resources and infrastructure)*
    - *Concerns on quality of services*
    - *Concerns on acceptability of population (trust of health insurance agencies and government, ability to pay, access, and culture)*
    - *Other concerns (e.g. security)*
    - *What strategies can address your potential concerns*
    - Do you think the country will ever be ready for health insurance? *Why? If so, when and how? What are some ways to reduce high out of pocket payments by households for health services?*
  - If support, what are the major reasons? *Probe*
    - The same dimensions mentioned above.
    - What is the health insurance model that you think is best applicable in Afghanistan?
      - *Probe- who will finance (government, household, donors, or combination of them), who should manage the health insurance, who should be covered, what are services should be covered, who should provide services.*
    - What are potential challenges to implement your proposed health insurance? How to overcome them?
    - What is most important, in order to successfully implement health insurance schemes in Afghanistan?
- Under health insurance schemes, households have to contribution to a certain amount of payment annual. As a return, you and your family member can seek a wider range of services, including both outpatient services and the majority of inpatient services, without cost or at very low costs. Would you or anyone you know be willing to pay for health insurance?
  - *Probe – types of services, under what conditions (quality, availability, etc), why or why not? How much contribution? How would they contribute?*
